# Upcoming Revolutionary Paths in Preclinical Modeling of Pancreatic Adenocarcinoma

**DOI:** 10.3389/fonc.2019.01443

**Published:** 2020-01-22

**Authors:** Mirna Swayden, Philippe Soubeyran, Juan Iovanna

**Affiliations:** Centre de Recherche en Cancérologie de Marseille (CRCM), INSERM U1068, CNRS UMR 7258, Aix-Marseille Université and Institut Paoli-Calmettes, Parc Scientifique et Technologique de Luminy, Marseille, France

**Keywords:** pancreatic cancer, organoids, cell culture, genetically engineered mice, 3D culture, xenografts models, 3D bio-printing, organ-on-a chip

## Abstract

To date, PDAC remains the cancer having the worst prognosis with mortality rates constantly on the rise. Efficient cures are still absent, despite all attempts to understand the aggressive physiopathology underlying this disease. A major stumbling block is the outdated preclinical modeling strategies applied in assessing effectiveness of novel anticancer therapeutics. Current *in vitro* preclinical models have a low fidelity to mimic the exact architectural and functional complexity of PDAC tumor found in human set, due to the lack of major components such as immune system and tumor microenvironment with its associated chemical and mechanical signals. The existing PDAC preclinical platforms are still far from being reliable and trustworthy to guarantee the success of a drug in clinical trials. Therefore, there is an urgent demand to innovate novel *in vitro* preclinical models that mirrors with precision tumor-microenvironment interface, pressure of immune system, and molecular and morphological aspects of the PDAC normally experienced within the living organ. This review outlines the traditional preclinical models of PDAC namely 2D cell lines, genetically engineered mice, and xenografts, and describing the present famous approach of 3D organoids. We offer a detailed narration of the pros and cons of each model system. Finally, we suggest the incorporation of two off-center newly born techniques named 3D bio-printing and organs-on-chip and discuss the potentials of swine models and *in silico* tools, as powerful new tools able to transform PDAC preclinical modeling to a whole new level and open new gates in personalized medicine.

## Introduction

Pancreatic ductal adenocarcinoma (PDAC) is an exocrine malignancy which accounts for more than 90% of all cases of pancreatic cancer (1). It is a highly lethal disease exhibiting an extremely poor prognosis, where <7% of patients survive for 5 years after diagnosis (2). Currently, PDAC is the fourth leading cause of cancer related death and should become the second deadliest cancer by 2030 (3). This morbid outcome is attributed to diagnosis at late stages of the disease, its rapid dissemination and its high resistance to all conventional treatments (4). Additionally, most patients are diagnosed with metastases in liver and lung due to the absence of early screening tools, and succumb to the disease within 6–12 months thereafter (5). Surgery is potentially the only curative option available, however only 10–15% of patients are eligible for resection (6). Over the last 20 years only 3 major improvements have been introduced as treatment regimens for PDAC. In 1997, Gemcitabine emerged as an alternative to 5-Fluorouracil as a first-line therapy for PDAC, it improved overall survival by only few weeks (7). Next, Folfirinox treatment regimen (5FU, Leucovorin, Oxaliplatin, and Irinotecan) was applied in metastatic patients and contributed to a significant improvement in survival although with strong adverse side effects which is limiting its application to all non-operable patients (8). In 2013, Nab-Paclitaxel (Abraxane) in combination with Gemcitabine was approved as a first-line treatment for advance PDAC patients as it gives better efficacy than Gemcitabine alone though, with little increase of adverse effects (9). Despite of these humble progress steps, the dismal clinical situation of PDAC patients still resides, where the incidence and mortality rates are constantly on the rise. Therefore, there is an urgent need for developing new and more successful therapeutic strategies to treat PDAC patients as well as new early detection tools and diagnostic and prognostic markers.

**Figure 1:**
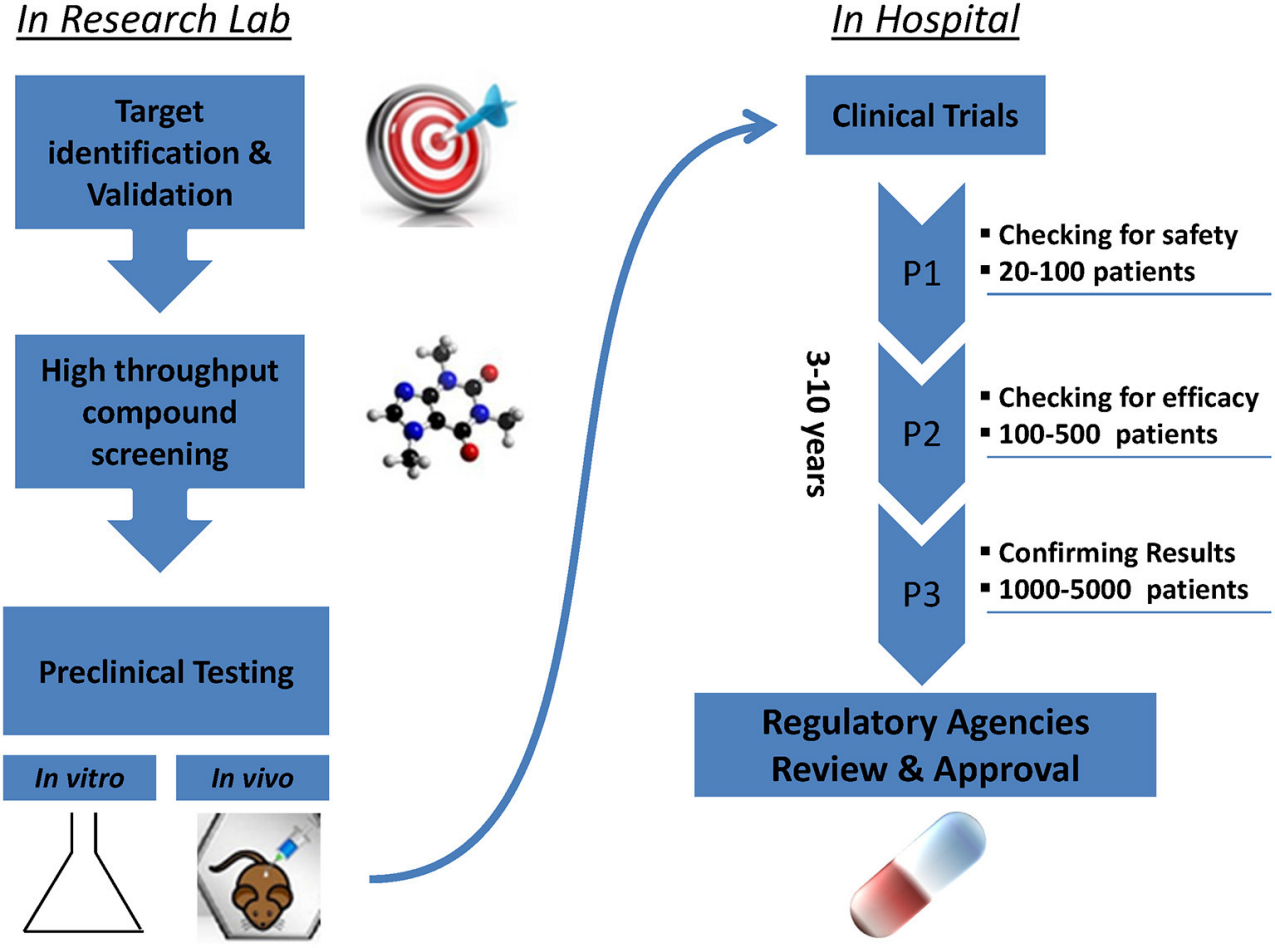
Steps of drug development from research lab to the patient's bed side.

**Figure 2:**
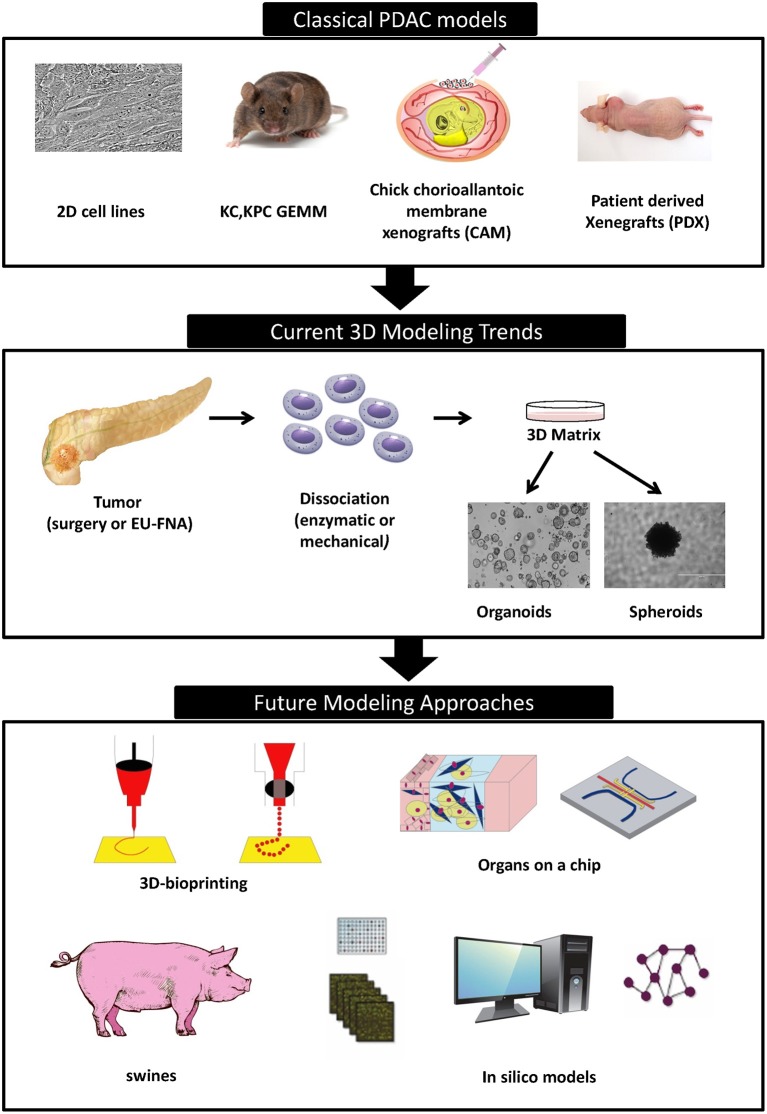
Timeline of different PDAC modeling approaches.

Promising new anti-cancer compounds are tested for their pharmacokinetics, safety, toxicity, and efficacy in a preclinical phase that acts as a bridge to the clinic (Figure 1). According to the Food and Drug Administration (FDA) regulations, an affluent preclinical testing must be completed before humans are exposed to the potential drug (10). The successful evaluation of therapeutics in preclinical settings highly depends on the accuracy, reproducibility and predictive ability of the *in vivo* or *in vitro* preclinical model used. New drug development programs usually take about 12 years to transfer a compound from experimental investigation to the patient bed side (Figure 1). Additionally, it is economically challenging with a cost as high as exceeding 1.2 billion dollars (11). It is also risky in terms of economic gain since 90% of tested drugs fail under clinical trials and only 10% could finally reach the market (12). This is mainly due to inconsistencies in the experimental model utilized leading to false uncertain conclusions. Several promising drug candidates failed clinical trials after a successful preclinical testing in animal models (13) due to genetic, immunologic, physiological, and metabolic differences between humans and mouse. In order to reduce the cost and the failure rate in clinical trials, solid trustworthy preclinical models must be developed for preclinical testing. These models must be reliable allowing the prediction of drug efficacy testing in humans and capable of closely recapitulating the true PDAC pathophysiology in human body. In this review we discuss the classical, existing, and the newly emerging preclinical model systems in PDAC research (Figure 2), highlighting the strengths, and weakness of each model. Also, we offer rationales for the implementation of innovative advancement technologies newly born in the field in PDAC research, aiming to create perfect modeling approaches to ensure success of cancer therapeutics in clinical settings.

## Classical Preclinical Models in PDAC Investigation

Traditional model system such as 2D cell lines, genetically engineered mice, and xenografts have shaped our current knowledge of PDAC pathology. However, the clinical relevance of these models have always been questioned. To date, the ability of these models to faithfully reflect the exact functional and structural properties of the tumor is still an unmet need. Several advantages and disadvantages characterize these models. A growing body of data urges us to develop novel preclinical testing models to bypass the pitfalls existing in current fundamental ones, able to better predict the success or failure of chemotherapeutic agents undergoing clinical trials.

## PDAC Cell Lines

Human derived cell lines are the most widely used models to study the biology of cancer. The first human pancreatic cancer cell line was generated in 1963 (14), and then many PDAC cell lines from human or murine tumors have been produced. Human cell lines are easy to manipulate, they can grow indefinitely at low cost and are suitable for high throughput pharmacological screening and genetic testing. However, key limitations exist within this model. First, most cell lines are derived from resected tumors, and since most PDAC patients are ineligible to surgery, then PDAC cell lines are derived only from a small subset of patients and doesn't reflect the heterogeneity found across PDAC tumors (15). Second, the *in vitro* culture of normal pancreatic ductal cells is a rather difficult task, thus the comparison between normal and PDAC cells is almost impossible (16). Third, the repeated passaging of cell lines results in a genetic drift and culturing cells as monolayers in medium containing serum was shown to promote the loss of p53 function and subsequent genome instability (17). Furthermore, several studies reported significant differences in expression profiles of cell lines as compared to patient primary tumors or xenografts (18). Finally, this model is not a faithful recapitulation of the histological and biological complexity of tumor, due to the lack of tumor microenvironment mainly composed of ECM (extracellular matrix) components and several cell types such as fibroblasts, nerves, immune cells, adipocytes, and endothelial cells (19).

## Genetically Engineered Mouse Models (GEMM)

Genetically engineered mice are designed by inducing specific mutations in oncogenes and/or tumor suppressor genes associated with PDAC in the mouse genome. This was firstly accomplished by introducing activating mutations in *KRAS* gene specifically in the pancreas which resulted in the formation of PanIN lesions capable of evolving to PDAC. The most well-studied GEMM of PDAC is surely the KPC one, which contains mutations in KRAS and TP53, both driven by the pdx1-Cre transgene which specifically expressed the Cre recombinase in all cells of pancreas starting from an early phase of embryonic development (20). In KPC model, tumors develop spontaneously with a dense desmoplasia and poor vasculature, similarly to human PDAC, thereby preserving the dynamics of tumor microenvironment (21). Therefore, it is a smart tool to study interactions between tumor and stromal cells in addition to disease progression from early stages of PanIN to primary and metastatic tumor. Moreover, GEMM intact immune system allows the study the immune response in PDAC and test novel immune-therapies (22). This model allowed scientists to reveal the complex balance between pro-malignant and tumor suppressive properties of tumor stroma (23, 24). Recently, these models were also employed to study the influence of microbiota composition and its impact on tumor development and patients survival, mainly by interacting and influencing the immune system (25, 26). GEMMs have led to pivotal strides in understanding PDAC pathogenesis by confirming causative roles of several mutant genes previously identified through human PDAC sequencing, in addition to identifying remarkable therapeutic targets such as MEK, PI3K, autophagy, and Notch pathways (27, 28). However, several pitfalls weaken this model: it is expensive due to the need of highly sophisticated imaging systems to monitor abdominal tumor growth within mice bodies. Also, it is labor intensive and time consuming to breed large number of mice colonies. Additionally, gene mutations are introduced into the germ line of the mouse, whereas they are occurring somatically and sequentially in human tumors. Several discoveries in murine models were not reproducible in human settings. For example, the anti-hedgehog therapy showed promising results in mice but failed to provide any clinical benefit in phase II study, suggesting that these models may lead to erroneous conclusions (21, 24). This can be attributed to interspecies differences in drug metabolism (29), immune function (30), and telomere activity (31) between mice and humans. Murine models have fewer mutations and less genetic complexity than human tumors. Moreover, the degree of aneuploidy in human tumors results in great variety of inter-tumoral gene modifications, in a totally different manner than it occurs in mouse (32). Taken all together, these species-related differences restrict the capacity of these models to predict the real therapeutic response of PDAC patients in clinical trial.

## Xenografts Models

These methods involve the engraftment of human cell lines (cell line derived xenografts or CDX) and human tumor fragments (patient derived tumor xenografts or PDTX) into immune-compromised mice.

### Cell Line Derived Xenografts (CDX)

One solution to address many of the drawbacks of 2D cultured cell lines is to transplant cell lines into immuno-deficient murine models to create a cell line derived xenografts either subcutaneously or orthotopically into the mouse pancreas. Pharmaceutical companies often use subcutaneous xenografts for drug testing due to their low cost, feasibility of rapid screening for efficiency and toxicity, and simplicity of tumor size assessment. On the other hand, orthotopic xenografts are expensive with an obligation of sacrificing the animal to detect drug response (33). However, this model is lame in predicting clinical outcomes, since xenograft murine model are immune-compromised and tumors are not subjected to pressure of immune system. Unfortunately, this model is non-feasible for evaluating immuno-therapies which is a serious need (34). Another limitation to this model is the selection of most aggressive and rapidly dividing cellular clones causing them to grow in homogeneous masses with a limited stromal infiltration, thus failing to mimic the exact morphology of the primary PDAC tumors (35). Additionally, these models are made from a limited number of cell lines therefore it fails to represent genetic and phenotypic heterogeneity existing in PDAC. Accumulating evidences show a moderate predictive values of this model and low correlation between data obtained from xenografts and the efficacy in clinic (36). For example Bruns et al. found that combining Gemcitabine with cetuximab (an EGFR inhibitor) induced 85% regression in a cell line xenografted, while proven ineffective in phase III clinical trial (37).

### Patient Derived Tumor Xenografts (PDTX)

PDTXs are made by transplanting a piece of patient's tumor tissue derived from surgical resection or from tumor biopsies in immune-deficient mice. PDTXs retain the morphological characteristics of the primary tumor as well as its metastatic potential (38, 39). Importantly, PDTXs mirror response of human patients to chemotherapy (40). A study subjected 4 PDAC-derived PDTXs to 63 different drugs in different combinations to determine the most effective ones against each PDTX. While three out of the four PDTXs were sensitive to gemcitabine, one showed sensitivity to mitomycin C as well as cisplatin. The patient corresponding this PDX didn't benefit from Gemcitabine and was treated with mitomycin C and then with cisplatin. This patient was still free of disease even 50 months after diagnosis (41). Remarkably, our lab has carried out the transcriptomic analysis of several PDTX-derived from pancreatic cancer and identified specific gene signatures able to predict sensitivity to several anti-cancer drugs and clinical outcome of PDAC patients (42). For example, after performing transcriptomic profiling of 55 PDTXs, we were able to demonstrated that PDTXs with MYC-high signature are more sensitive to the BET bromo-domain inhibitor JQ1 than the PDTXs with MYC-low activity (43). Despite the promising potential of PDTX as preclinical drug testing platforms, several concerns need to be addressed such as, for example, the fact that PDTXs don't fully replicate stromal compartment of PDAC or host immune system (44). The use of immune-compromised mice limits the ability of using PDTX to examine responses to new immune-therapies. Moreover, the generation and maintenance of large colonies of immune-compromised mice for PDTX passages is extremely costly. Another drawback is that PDTX take up to 6 months to grow and waiting this long is simply untenable for most PDAC patients. To ensure a successful transplantation, a large amount of tumor tissue after surgical resection or biopsies for non-resectable patients is needed. More recently, we reported an efficient protocol to obtain PDTX directly from material recovered from endoscopic ultrasound-guided fine needle aspiration (EUS-FNA) (45). This technical improvement must be considered as a major progress since after that, virtually all human tumors can be grown as PDTX and therefore studied. An important disadvantage of this model is that it can't precisely represent the heterogeneity of PDAC since it has been shown that most aggressive phenotypes are favored to propagate within the mouse model (46). Finally, xenografts derived from PDAC patients become infiltrated with murine stroma (45, 47), and most of human stroma that does not grow is replaced with murine cells after two or three passages (48). This mismatch between human tumor cells and mouse stroma must be taken into account when evaluating studies using PDTX model which can become an advantage as it enable the study of the interaction and communication between these two cellular compartments (45).

Creative approaches are being developed to bypass the limitations of PDTX model. One way is the co-implementation of stromal cells derived from patients (cancer associated fibroblasts, CAFs) that could prevent the invasion of the murine microenvironmental components thereby configuring the xenograft in a human manner as much as possible. In this way, a recent study showed that orthotopic co-transplantation of patient derived CAFs along with Capan-2 pancreatic cancer cells in NSG (NOD scid gamma mouse) mice revealed the tumor and metastasis-promoting ability of these patient derived CAFs (49). Importantly, CRISPR/Cas9 technology permitted the creation of syngeneic and humanized mice (50). An approach that will certainly benefit from the last advances of genome editing such as prime editing which has been described very recently and brings an important enhancement of the efficacy and specificity (51). Syngeneic/allografts transplant models are developed through the transplanting of cancer cells or solid tumors derived from the same genetic strain of mice (50). This method prevents the rejection of the transplant by the host's immune system, hence avoiding the need for immune-compromised mice, and allows the possibility of investigating immune-therapies in these models. But the major shortcoming of syngeneic mice is that the tumor cells are rodent and therefore doesn't fully mimic human PDAC (50). Syngeneic mice were successful platforms for identifying the currently approved immunotherapies such as checkpoint blockers including anti-CTLA4 (52), anti-PD-1 and anti-PDL-1 (53). Another approach is the production of humanized mice having mutations in IL-2 receptor gamma chain which allows the engraftment of hematopoietic stem cells and subsequent development of human immune systems. These mice are used to study human hematopoiesis and immunity, and can also be used to study cancers development with an immune component (54). Unfortunately, humanized murine models cannot fully replicate the human immune system due to a limited lymph node development, the HLA super families, and the appropriate immune cell trafficking (55). To date, there are limited trials of testing checkpoint inhibitors combinations in humanized mouse models. Sanmamed et al. used humanized mice model mice to test the efficacy of the combining a immune checkpoint inhibitor (nivolumab, anti-hPD-1) with an immune-stimulatory monoclonal antibody (urelumab, anti-hCD137). This study was an initial proof of concept that combination regimens can be modeled in humanized mice model. Authors were able to detect the expression of PD-1 and CD137 (Tumor Infiltrating Lymphocytes: TILs) and PD-L1 (tumor cells and antigen presenting cells) even when differences in tumor sizes were not significantly different upon the use of combination therapy vs. mono-therapy (56).

### Chick Chorioallantoic Membrane Xenografts (CAM)

Chick chorioallantoic membrane is a highly vascularized extra-embryonic membrane connected to the embryo through a continuous circulatory system. It is considered easily accessible for experimental manipulation such as intravenous injection of therapeutic compounds and visualization of local response (57). It is not immune-competent until day 18, this feature makes it ideal for grafting foreign tissues without rejection ahead of this time (58). The CAM is a low cost model allowing the preclinical screening to assess the efficacy of large number of anti-cancer drugs on tumor growth (59). It is particularly faster than most mammalian models as tumor grafts become vascularized by chick vessels 2–5 days after inoculation (60). The typical CAM assay involves lowering the membrane by forming an air pocket between the shell membrane and the CAM itself, then tumor cells are grafted as an inoculum introduced through a small window made in the shell above the CAM at day 9 of embryonic development and tumors are harvested at day 16 (61). Tumor cells can be visualized in the CAM assay using diverse techniques like *in vivo* videomicroscopy, detection of human urokinase plasminogen activator, GFP-labeled cells, PCR amplification of human specific sequences, PET/CT imaging, viral nanoparticles, and immunohistochemistry (61). Sys et al. clearly demonstrated that tumor fragments grafted in the CAM retained the morphology of primary tumor. However, tumor-associated stroma from the human samples was largely replaced by stroma coming from chicken in the grafts (62). A potential limitation is the arising of a non-specific inflammatory response after 15 days of incubation (63). A study by Rovithi et al., provided the first evidence that primary PDAC cells transduced with firefly luciferase can form tumors on the CAM, retaining several histopathological and genetic/epigenetic characteristics of primary tumors. They also used this model for testing the modulation of key miRNAs and the activity of Gemcitabine and Crizotinib on CAM tumors. Interestingly, they showed that combined treatment resulted in 63% inhibition of tumor growth and was associated with a reduced expression of miR-21 and increased expression of miR-155 (64).

CAM is a relatively rapid, straightforward, and economical model that permits screening of a large number of pharmacological agents in a short time range. Fortunately, this model doesn't require the administrative procedures to obtain the pre-approval of animal experimentation by the ethics committee, since the chick embryo conventionally is not considered a living animal until day 17 of development. Within the CAM the vascularization network and tumor cells development occur in a quick manner, this enable the researcher to closely observe and track the real time morphological changes of cancer cells in its microcirculation (63). However, several issues were raised concerning this model, one of them being the scarce availability of commercial reagents (ex: primers, antibodies, metabolic kits, etc.) suitable for application in avian species. Another concern was related to the reliability of angiogenesis studies carried out within this platform. Timing must be an essential factor to be considered when planning angiogenesis analyses, this due to the difficulty to distinguish between the real neovascularization from a false increase in vascular density caused by remodeling of the pre-existing vessels (65). Finally, CAMs are extremely sensitive to stress induced by environmental factors such as oxygen, tension, pH, osmolarity, and keratinization levels, this may complicate the sealing process of the opening made within the shell (66).

## 3D Organoids: The Current Growing Trend

3D culture methods are promising tools to better mimic the tumor biology found *in vivo*. The main goal of 3D culture is to avoid the cells attachment to the bottom of culture dish, either by keeping cells in suspension or by culturing cells in the presence of a special matrix. 3D systems increase the number of cell-to-cell interactions and resemble more closely the architectural organization of cells *in vivo*. 3D cultures derived from monolayer cell lines are referred to as spheroids that share common characteristics with cells *in vivo* including production of ECM, increased chemo-resistance and appearance of polarized cells junctions. However, as previously described, the use of monolayer cell lines as a starting material is a limiting point to this model and practically decreases its *in vivo* relevance (67, 68). On the other hand, a group of cells growing in 3D culture derived directly from primary tissues, embryonic stem cells or pluripotent stem cells are termed organoids. They possess a self-renewal and self-organizing capacity, and maintain similar morphologies and functionalities as the original tissue *in vivo*. Interestingly, organoids can be cryo-preserved and replicated passaged indefinitely keeping their genetic stability (69). This 3D system is amenable for genetic, transcriptomic, proteomic, and biochemical analysis. Briefly, 3D organoids are created by the enzymatic and/or mechanical dissociation of the tumor (or normal tissue) into small pieces that are then embedded in a specialized matrix, usually collagen, or matrigel, with addition of specific growth factors and differentiation modulators to furnish mesenchymal-like signals, such as EGF, FGF10 (mitogens), Rspo1 (to enhance Wnt signaling), Noggin, Wnt3a, nicotinamide, N-acetylcysteine, gastrin, and A83-01 (Alk inhibitor). Prostaglandin E2 is required in the case of normal pancreas human organoids (70). In 2015, the first human and murine PDAC organoid model was established; it recapitulated successfully the physiological and morphological similarities to normal and tumor tissues from mice and human. These organoids expressed ductal epithelial markers and lacked genes representing acinar and endocrine lineages. Interestingly, when these organoids are transplanted orthotopically in to immune-deficient mice they generate pre-invasive lesions similar to PanINs able to progress in to PDAC and metastasis, thus representing an appealing model for studying PDAC progression. Additionally, it has been demonstrated by gene expression and proteomic analysis in murine pancreatic 3D organoids that both transcriptomic and proteomic profiles correlated with the original primary tumor (71). 3D organoids can potentially advance personalized medicine for PDAC; they form reliable platforms for evaluating potential diagnostic markers and wide anti-cancer drug screening. Organoids can be generated also from biopsies refined via EUS-FNA, thereby representing closely the heterogeneity of different PDAC stages and clinical conditions from both resectable and non-resectable patients. In a clinical trial setting, patient individual tumors can be utilized to form 3D organoids. A subsequent large-scale drug testing can be conducted within the next weeks after receiving the biopsy of a given patient. Such testing aims toward the identification of individual therapeutic sensitivities based on genetic alteration signatures and/or drug responses in organoids, in order to determine second-line therapies for prolonging survival and enhance quality of life in PDAC patients when the response to first-line therapy is minimal or strongly reduced. To fulfill the aim of analyzing potential biomarkers and stratifying patients based on their genetic profiles and drug response, bio-banks of 3D organoids from surgery or endoscopic ultrasound are being created in our laboratory and validated as a tool of clinical interest (72, 73). However, it is worth noting that this model is facing critical challenges to be routinely applied in clinic, since it is an expensive, time consuming, and basically lacking the components of microenvironment and immune cells normally found *in vivo*. Additionally, all the added external factors which are not necessarily present in parental tumor may lead to artifactual findings. Tsai et al. tried to surmount this limitation by co-culturing primary pancreatic cancer cells along with immune cells and other types of stromal cells to investigate the tumor-stroma or the tumor-immune cells interaction and assessment of immune-therapeutics using organoid models (74). Moreover, heavy chemotherapeutic pre-treatment prior to surgery or biopsy and low cancer cells content of biopsy can lead to failed organoid culture. This is only the starting point of 3D organoid model and it is crucial to standardize protocols used for isolation and passaging of 3D organoids, since currently this technique is of heavy cost and technically challenging since it requires precious patient samples. Importantly, more studies will determine if these biopsy-derived organoids faithfully represent the genetic heterogeneity and therapeutic efficacy profile of the entire primary tumor and the extent of translating its outcomes in patients.

## The Dawn of Next-Generation Preclinical Models in Oncology

To date the major obstacle toward creating a perfect pre-clinical model with an absolute reliability is the poor representation of tumor microenvironment, where many studies well-demonstrated the strong influence of micro-environment components on therapeutic outcome. However, recent innovative advancements, such as 3D Bio-printing and organs-on-a-Chip, are able to meticulously simulate this tumor micro-environment. Such revolutionary models can open up a new frontier in oncology research and accelerate the development of new cancer therapeutics.

### 3D Bio-Printing

3D bio-printing technology generates bio-printed tissues and organs, where 3D bio-printers deposit several types of co-cultured cells in single spatial arrangement matching the natural architecture of native tissue. 3D bio-printers use various types of cells in the form of bio-inks that are mainly composed of cells suspended in a bio-compatible gel-like material. These bio-inks are deposited on a 3D scaffold after which it is gelled by polymeric inter-linking using photo or thermal activation (75). The 3D organ scaffolds are solid surfaces made up of non-toxic bio-compatible materials similar to the human ECM such as natural polymers (alginate, gelatin, collagen, chitosan, fibrin, hyaluronic acid, etc.) or synthetic molecules such as polyethylene glycol (76). Non-invasive imaging methodologies, such as computed tomography, magnetic resonance imaging, computer aided design and computer aided manufacturing tools, and mathematical modeling, are used to digitize and model the architecture of the tissues and organs in order to generate a mimicking 3D scaffold. Then, digital images are used to print tissues and organs using techniques such as laser-assisted printing (77), micro-extrusion (78), and inkjet (79). 3D bio-printed tissues or organs are novel platforms for pre-clinical anti-cancer drug testing. Organovo is a medical company in its early stages that designs functional 3D human tissues and organs for medical research aiming to accelerate the preclinical and clinical therapeutics testing at low cost and no potential risks for living patients. These bio-printed tissues and organs provide a similar micro-environment to that of native organ in the body, conserving the interaction of cells with environmental factors and biology of ECM. Importantly, this may reduce the chances of failure and costs in human clinical trials (80). 3D bio-printing technology can be utilized to produce 3D tumor models for the study of cancer biology. Several approaches such as cell seeding 3D scaffold, hydrogel embedding, multi-cellular spheroids, cell patterning, and micro-fluidic chips have been exploited to build up a 3D tumor model *in vitro* (81). Zhao et al. used HeLa cells in gelatin/alginate/fibrinogen hydrogels to bio-print 3D *in vitro* models of cervical tumors. This study revealed the increased expression of matrix metalloproteases and chemo-resistance in 3D printed tumor models when compared to 2D cell culture model (82). Similarly, it is possible to generate bio-printed PDAC tumors that can be exploited as a more transparent model of drug testing. This technology is still in its infancy and more studies must be carried out to implement this valuable platform in the preclinical modeling of PDAC as well as other tumor types. However, 3D bio-printed tumor models are a faithful match to organ *in vivo* and have the potential to revolutionize the entire oncology research and drug discovery.

The key advantage of bio-printing cancers cells in 3D lies it its ability to model the tumor microenvironment *in vitro* with highest fidelity, thereby offering a better representation of tumor formation, progression, and response to anti-cancer drugs. Several studies proved the contribution of microenvironment's components in chemoresistance. Therefore, utilization of this platform is ideal for personalized drug screening procedures (83). Despite the advancement, challenges lie ahead of this newly born technology. Current light based bio-printer can produce bio structures at a microscale resolution, where there is a great demand to achieve more sophisticated single cell structures such as networks of blood capillaries. Another factor to be enhanced is the speed of printing process since the viability of cells within the bio-ink decreases as printing time increases especially for cell types with a high metabolic profile like muscle cells for example. Additionally, no real studies investigated the effect of bio-printers on the molecular features of the cells such as gene expression or other functional aspects. Another issue lies in scaling up this technique to generate large amounts of bio-printed tumors or tissues for clinical and commercial applications. Moreover, to date, the efforts for incorporating patient's primary cells in to this platform are scarce and poorly developed. Future work is greatly needed to standardize the protocols of this technique to overcome these pitfalls, and therefore offering the best version possible for modeling diseases with accuracy. This technique, with its great innovation, holds the potential to the experimental bridge to novel clinical regimens (84).

### Organ-on-a Chip

Organs on chips are micro-fluidic devices used for culturing cells, composed of plastic, glass, or polymers such as polydimethylsiloxane with hollow micro-channels populated with viable cells. These micro-fluidic devices can form tissue chips made up of a single channel lined by cells from one tissue type or organ chips with higher complexity. Organ chips combine two or more types of tissue interacting directly across a porous membrane coated with ECM or separated by an ECM gel filling the micro-channels. Cells within this system are nourished with flowing culture medium through the endothelium-lined vascular channels (85). Cultured medium can be replaced with blood for few hours of culture (86). Additionally, these devices may contain hollow side chambers of cyclic suction for the application of rhythmical stretching and relaxing of tissues interfaces and therefore replicating the organ specific mechanical signals. These organ chips devices can reproduce the organ level response to drugs (87) and mimic several types of organ specific diseases including cancer. Such technology is applied in modeling basic hallmarks of cancer including tumor growth, progression from early to late stages, invasion, angiogenesis, EMT (epithelial to mesenchymal transition), and metastasis (88). Organ-on-chips were used to generate *in vitro* human orthotopic cancer model in non-small cell lung carcinoma (89) and multiple myeloma (90). The major breakthrough was implementing organ-on-chips approach into modeling responses to anti-cancer therapies. One study used a micro-fluidic device created with various oxygen gradients within, to test the sensitivity of A549 lung alveolar epithelial cancer cells to tirapazamine (that is converted in to active free radical under hypoxia). The results illustrated an increased drug efficacy under low oxygen gradient (91). Another study utilized an organ-on-chip system containing 3D chambers seeded with A549 lung cancer cells connected to another chamber containing cultured CAFs and attached to a linear concentration gradient generator. Authors showed that secretion of HGF by CAFs could inhibit paclitaxel-induced death in lung cancer cells (92). Such studies are excellent examples of how precise control over chemical gradients is made feasible using this technology. In an early attempt to apply this technology in the field of personalized medicine, a team cultured lung cancer cell line, a mixture of lung cancer and stromal cell lines, or cells from fresh lung cancer tissues in 3D gels within a micro-fluidic device and treated them with different concentrations of chemotherapeutic drugs generated on-chip using a micro-fabricated concentration gradient generator. The sensitivities to different anticancer agents (Gefitinib or Cisplatin) were determined in parallel, and the doses of single drugs and combinations were optimized in eight patients (93). Another study aimed at screening for drugs able to prevent tumor dissemination. To this end, human lung adenocarcinoma or bladder carcinoma cell lines were embedded in a collagen matrix in one channel, and HUVECs (Human umbilical vein endothelial cells) were cultured in another channel where they formed vessel-like structures. Then, the entire culture was treated with different drugs known to affect EMT pathways. These drugs significantly reduced expression of EMT markers when tumor cells were cultured alone. However, these effects were diminished in the co-culture with HUVECs. These results highlighted the role of tissue-tissue interfaces and of an *in vivo*-like microenvironment in the evaluation of anticancer agents (94). A pioneering study by Beer et al. provides evidence that micro-fluidic chips can be applied to culture PDAC cells, while maintaining their viability, proper morphological appearance, and growth characteristics. This micro-fluidic chamber platform was used to detect the drug response of PDAC cells to the chemotherapeutic agent Cisplatin (95). This technology recapitulate successfully the tissue-tissue interface and the physiological microenvironment that are crucial for reconstituting the complex organ level architecture and function. Such privileges are not available in 2D and 3D *in vitro* models. Also, it can be exploited to gain new insights into fundamental processes involved in cancer biology and therapeutic targeting.

### Porcine Cancer Models

Incorporating the use of swine as a large animal model in cancer research is of a important potential benefits. Swine models have several advantages as they share tremendous similarities with humans on the level of genetics, epigenetics, anatomy, size, metabolism, and pathology in addition to their reduced expenses compared to other primate models (96). Hence, disease modeling in these animals can better portray cancer development and progression as seen in humans.

Thanks to advances in genetic engineering, genetically modified pig cancer models were created and such models are able to respond to therapy in a similar fashion to humans in randomized trials (97). Current porcine models which are used for cancer research include the APC^1311^ model of familial adenomatous polyposis producing polyps but no tumor (98), the heterozygous TP53 knockout model of osteosarcoma (99), and a chemically induced porcine HCC (hepatocellular carcinoma) model (100). The Oncopig Cancer Model (OCM) is a novel transgenic swine model representing the next generation large animal platform for tumor studies in oncology. Like in mice, this model uses a Cre recombinase expression to induce the expression of heterozygous KRAS^G12D^ and TP53^R167H^ transgenes (101). A porcine PDAC model is being developed using the OCM. Induction of exocrine and neuroendocrine pancreatic cancer types was demonstrated in the OCM through delivery of AdCre (Cre expressing adenovirus) in to the pancreatic duct. This method led to the development of an invasive PDAC tumor with similar histological hallmarks to human PDAC, with dense fibroblastic stroma and acinar to ductal metaplasia (102). Such PDAC porcine model can aid in better understanding of early events of carcinogenesis and facilitating earlier detection, in addition to investigating new surgical strategies as well as studying the potential of nanotechnology and localized drug delivery approaches for pancreatic cancer. Pigs have been used as a preclinical model for drugs toxicology prior to human studies. The size and easy handling of pigs allow drug administration in a similar manner to human patients. It is possible to perform longitudinal blood sampling in order to assess drug exposure kinetic data and metabolism over long periods of time (103), since studies have shown similar kinetic response to humans that can't be modeled in another animal (104). It is worth noting that OCM is considered ideal for identification of putative blood biomarkers and prognostic indicators. Porcine models are newly emerging approaches with a great potential to drive translational cancer research toward success and address the unmet clinical needs. Importantly, OCMs can be further applied to model additional cancer types such as leukemia, lymphoma, and other hematological cancers.

### *In silico* Models

With the advancements of experimental tools for measurement of cancer genome, transcriptome, and proteome such as: microarrays platforms, next-generation sequencing, and mass spectrometry, high-throughput cancer biology data are being generated in conjunction with analytical computer-based technologies. Such information rich data permit the construction of highly sophisticated computational *in silico* models that aid in biological discovery and personalized medicine. Key *in silico* models are being utilized in oncology. They include the creation of cancer statistical models which rely on signatures of genes expression and mutation, of perturbed molecular pathways and networks, of alterations of biochemical, metabolic, and signaling, and the modeling of the interactions between tumor cells and their microenvironment (105). Cancer Statistical models based on gene mutations signatures are applied for diagnosis of cancer subtype and stratification of tumor grade as well as predicting therapeutic outcome. Relapse and overall survival were successfully modeled in non-small lung carcinoma (106), pediatric leukemia (107), and breast cancer (108). Additionally, models of transcriptional classifiers have been used to anticipate tumor response to chemotherapies in breast cancer (109, 110), colorectal cancer (111), and ovarian cancer (112). *In silico* approaches offer a tremendous potential in drug discovery. This process starts with target identification through chemo-informatics tools such as chemical structure similarity searching (113), data mining/machine learning (114), panel docking (115), and bioactivity spectra based algorithms (116). Once the target is identified and validated, *in silico* tools can initiate drug design process by the structure based or ligand based computer aided drug design (117). The Team of Ma et al. developed a computational model capable of predicting drugs to treat pancreatic cancer. Seven drugs were identified using this model, three of which are supported by literature findings and three are experimentally validated by cytotoxicity assays using cell lines (118). Moreover, *in silico* models are used for toxicity assessments on the level of liver, gastrointestinal tract, and blood-brain barrier (119, 120). Such computational models aim to understand the side effects of drug candidates from molecular changes to phenotypic manifestations. It has been proven effective for optimizing dose and minimizing costly phase I/II clinical trials (121, 122). Despite of confronting substantial experimental and analytical obstacles, these models are promising reliable digital representations of cancer, with the purpose of early diagnosis, prognosis, and new therapeutics innovation without exposing patients to risks.

## Conclusion

Traditional preclinical models contributed to major advancements in our understanding of PDAC so far. However, they are still far of being able to ensure translational success. This is mainly due to poor representation of microenvironment of human neoplasia and absence of immune system, or the reaction of the full organism. These factors were proven to strongly affect clinical outcome and drug responses to treatments. New cancer therapeutics must be accelerated through the implementation of new sophisticated technologies able to simulate all the characteristics of PDAC within the human body (Figure 2). Such technologies will create a progressive preclinical drug screening leading to authentic conclusions highly reproducible in clinical trials and ensuring the benefit of participating patients.

## Author Contributions

MS, PS, and JI wrote, read, and approved the final manuscript.

### Conflict of Interest

The authors declare that the research was conducted in the absence of any commercial or financial relationships that could be construed as a potential conflict of interest.
